# Drug–drug interactions: A descriptive analysis of FDA adverse event reporting system

**DOI:** 10.1097/MD.0000000000044606

**Published:** 2025-09-19

**Authors:** Abdullah Alahmari, Sarah Fatani, Nehad Ahmed

**Affiliations:** a Department of Clinical Pharmacy, College of Pharmacy, Prince Sattam Bin Abdulaziz University, Al Kharj, Saudi Arabia.

**Keywords:** adverse events, drug interactions, FAERS, reporting

## Abstract

A drug–drug interaction (DDI) occurs when the administration of 1 medication alters the effects of another and is a potentially avoidable source of medication-related harm. This study aimed to characterize the DDI reports submitted to the Food and Drug Administration Adverse Event Reporting System (FAERS). The current study is a retrospective study that used the FAERS. It includes DDI data filed before 1st of April 2024. In all, 167,065 cases were reported to the FAERS. 153,383 of these events were classed as significant interactions, and 14,723 resulted in death. The most commonly reported medications were warfarin (4.33%), aspirin (4.19%), sertraline hydrochloride (3.25%), tacrolimus (3.02%), simvastatin (2.93%), and fluoxetine hydrochloride (2.84%). Healthcare practitioners should educate their patients about potential adverse events, such as drug interactions, and how to avoid them. These findings have implications for clinical practice, pharmacovigilance initiatives, and public health measures focused on reducing the risks of drug interactions and enhancing patient safety.

## 1. Introduction

The age of fast progress in pharmacotherapy has undoubtedly revolutionized patient healthcare.^[1]^ Through innovative medications and treatments, individuals have experienced remarkable improvements in their quality of life and longevity.^[1]^ These advancements have enabled patients to manage chronic conditions more effectively, alleviate symptoms, and even prevent diseases altogether.^[1]^ However, pharmacotherapy’s rapid evolution alongside these benefits presents a dual challenge.^[2]^ Despite its potential to save lives, it also carries the risk of severe and sometimes fatal adverse effects.^[2]^ Research indicates that pharmacotherapy-related adverse events contribute to a significant portion of hospital admissions, comprising approximately 3.6% of cases.^[3]^ This underscores the critical importance of vigilant monitoring, careful prescription practices, and ongoing research to mitigate risks and maximize the benefits of modern pharmacotherapy.^[3]^

Drug–drug interactions (DDIs) represent a substantial risk factor for adverse outcomes within pharmacotherapy. These interactions occur when 2 or more drugs interact, altering the effectiveness or toxicity of 1 or more medications.^[4]^ The spectrum of harm resulting from DDIs is broad, encompassing anything from mild symptoms that may not significantly impact treatment to severe adverse effects that can lead to hospitalization or even death.^[5]^ The severity of these interactions can be influenced by various factors, including the pharmacological properties of the drugs involved, the dosage and duration of treatment, as well as individual patient factors such as age, underlying health conditions, and genetic predispositions.^[5]^ Given the potentially serious consequences of DDIs, healthcare providers must remain vigilant in assessing and managing these interactions to minimize risks and optimize patient outcomes.

In 2017, the World Health Organization unveiled its Third Global Patient Safety Challenge, titled “Medication Without Damage,” with the ambitious goal of reducing severe preventable medication-related harm by 50% worldwide between 2017 and 2022.^[6,7]^ Among the various factors contributing to medication-related harm, DDIs stand out as a potentially preventable cause.^[8]^ DDIs occur when the effects of 1 drug are altered by the presence of another, leading to adverse outcomes.^[8]^ In this interaction, the drug affected by the interaction is often referred to as the object, while the drug influencing its effects is termed the precipitant.^[9,10]^ Precipitant drugs can modify the action of object drugs through diverse mechanisms, including pharmacokinetic and pharmacodynamic processes.^[9]^ Pharmacokinetic interactions involve changes in drug absorption, distribution, metabolism, or excretion, while pharmacodynamic interactions affect the drug’s mechanism of action or physiological response.^[9]^ Understanding and managing these interactions are crucial steps in promoting medication safety and achieving the World Health Organization’s ambitious patient safety goals.

The voluntary utilization of pharmacotherapy often leads to the simultaneous administration of multiple medications in a patient. While not all DDIs result in harm or severe health consequences, some can lead to diminished therapeutic effectiveness, adverse drug reactions (ADRs), or toxicity.^[11]^ These consequences can manifest in various clinical indications, including the failure to achieve therapeutic goals, deterioration of the patient’s condition, or in extreme cases, mortality.^[11]^ Moreover, DDIs can substantially impact healthcare service utilization, notably contributing to increased hospitalization rates.^[11]^ Beyond their clinical ramifications, DDIs also exert significant economic effects, imposing additional financial burdens on individuals and healthcare systems alike.^[11]^ The recognition, assessment, and management of DDIs are therefore essential components of safe and cost-effective pharmacotherapy, underscoring the importance of comprehensive medication management strategies and interdisciplinary collaboration among healthcare professionals.

The Food and Drug Administration (FDA) has launched a new interactive, web-based dashboard search tool for its FDA Adverse Event Reporting System (FAERS) database, significantly enhancing accessibility to data concerning adverse reactions to medications and therapeutic biologic products reported to the FDA.^[12–16]^ FAERS stands as one of the largest federal databases, housing comprehensive records on prescription errors and adverse event reports submitted to the FDA.^[12–16]^ It holds considerable significance as a valuable resource for monitoring the effects of medications, as well as for identifying and evaluating adverse events that were previously unreported.^[17]^ Despite its recognized importance, there is a notable lack of data specifically focused on adverse event reports related to DDIs within the FAERS database. Therefore, to close this knowledge gap and advance our understanding of medication safety and possible interactions, the current study set out to give a thorough assessment of the reports of DDIs filed to FAERS.

## 2. Materials and methods

For this retrospective investigation, the researchers utilized the FAERS to conduct the analysis. The study involved a descriptive examination of all adverse events that had been reported and were linked to DDIs. By leveraging the extensive data available in the FAERS database, researchers were able to perform a detailed retrospective analysis to better understand the nature and characteristics of these drug interactions and their associated adverse outcomes. This approach allowed for a thorough exploration of the reported incidents, providing valuable insights into the prevalence, types, and potential impacts of DDIs on patient safety and healthcare outcomes.

To identify reports related to medication interactions within the FAERS, the researchers conducted searches using specific reaction terms rather than product terms. Five keywords were employed for this purpose: “drug interaction,” “labeled DDI issue,” “labeled DDI medication error,” and “potentiating drug interaction.” These targeted search terms were chosen to capture a comprehensive range of adverse events associated with DDIs. By focusing on reaction terms, the study aimed to systematically identify and include relevant reports detailing instances of medication interactions reported to the FAERS database. This approach facilitated the retrieval of data specifically related to drug interactions, enabling a more focused analysis of these critical safety concerns.

The analysis gathered all reports of DDIs that had been submitted to the FAERS by healthcare practitioners or other relevant personnel up until March 31, 2024. However, to maintain consistency and focus on preestablished data, any reports of adverse events or DDIs filed after March 31, 2024, were excluded from the research. This approach ensured that the study’s findings were based solely on data collected within a specific timeframe, allowing for a more coherent and reliable analysis of DDIs reported to the FAERS database.

In this current study, we conducted a descriptive analysis of DDIs reported to the FAERS. The investigation encompassed the frequency of these interactions, the specialized backgrounds of the reporters submitting the reports, the gender distribution of affected patients, their age demographics, and the medications most frequently implicated in these interactions. Descriptive statistics were utilized to summarize and report these findings and presented as frequencies and percentages.

## 3. Results

Until March 31, 2024, a total of 177,174 cases were reported to the FAERS. Among these, 154,547 events were classified as significant interactions, and 14,821 resulted in death. Notably, more than 19% of the reports were filed between 2021 and 2023, while 22.94% of the reports were submitted between 2018 and 2020 (Table 1).

**Table 1:** The years of reporting drug interactions.

Year	Number	Percentage
2021–2024	34,435	19.43%
2018–2020	37,919	21.40%
2015–2017	25,232	14.24%
2012–2014	18,542	10.47%
2009–2011	14,467	8.17%
2006–2008	10,413	5.88%
Before 2006	36,166	20.41%
Total	177,174	100.00%

Table 2 presents the age distribution of patients who experienced DDIs. The data reveals that most patients fell within 2 age groups: 18 to 64 years, accounting for 52.49% of cases, and 65 to 85 years, representing 36.77% of cases.

**Table 2:** The age of the patients.

Age category*	Number	Percentage
0-1 month	187	0.13%
2 months–2 years	755	0.55%
3–11 years	2814	2.05%
12–17 years	3432	2.50%
18–64 years	72,192	52.49%
65–85 years	50,569	36.77%
More than 85 years	7584	5.51%
Total	137,533	100.00%

* The age was not specified in 39,641 reports, so we excluded these report data in the table.

Table 3 displays the gender distribution of patients who experienced DDIs. The data indicates that a slightly higher proportion of affected patients were female, accounting for 52.77% of cases, while males represented 47.23%.

**Table 3:** The gender of the patients.

Gender*	Number	Percentage
Female	83,234	52.77%
Male	74,492	47.23%
Total	157,726	100.00%

* The gender was not specified in 19,448 reports, so we excluded these report data in the table.

Table 4 outlines the specialty of the reporters who submitted the reports on DDIs. The data indicates that most reporters were healthcare professionals, comprising 74.81% of submissions, while consumers accounted for 25.19% of reports.

**Table 4:** The specialty of the reporters.

Category*	Number	Percentage
Healthcare professional	124,877	74.81%
Consumer	42,050	25.19%
Other	3	0.00%
Total	166,930	100.00%

* The specialty was not specified in 10,244 reports, so we excluded these report data in the table.

Table 5 presents the outcomes of drug interactions reported in the study. These outcomes are died, disabled, hospitalized, life-threatening, and others. Died refers to fatal outcome directly or indirectly attributed to the drug interaction. Disabled refers to persistent or significant disability/incapacity resulting from the interaction. Hospitalized refers to interactions required inpatient admission or prolonged hospitalization due to the adverse effects. Life-threatening refers to immediate risk of death or serious harm necessitating urgent intervention. The data reveals that over 44% of the interactions resulted in hospitalization, indicating the severity of the adverse events experienced by patients. Additionally, 8.37% of patients died as a result of drug interactions, underscoring the significant impact of these events on patient safety and health outcomes. Furthermore, 9.27% of the interactions were classified as life-threatening, further highlighting the seriousness of the adverse effects associated with certain medication combinations.

**Table 5:** The outcomes of drug interactions.

Category	Number	Percentage
Died	14,821	8.37%
Disabled	4876	2.75%
Hospitalized	78,106	44.08%
Life-threatening	16,417	9.27%
Others	62,954	35.53%
Total	177,174	100.00%

Table 6 illustrates the priority assigned to the cases of drug interactions reported in the study. The data indicates that the majority of interactions were classified as expedited cases, accounting for 72.30% of reports. Additionally, a smaller proportion of cases, 2.91%, were classified as direct priority.

**Table 6:** The priority of the cases.

Category	Number	Percentage
Expedited	128,096	72.30%
Direct	5152	2.91%
Other	43,926	24.79%
Total	177,174	100.00%

Table 7 presents the most frequently reported drugs that interacted with other medications. The data indicates that the most commonly reported drugs that interacted with other drugs were warfarin sodium (4.32%), aspirin (4.20%), sertraline hydrochloride (3.24%), tacrolimus (3.09%), simvastatin (2.91%), fluoxetine hydrochloride (2.85%), clopidogrel bisulfate (2.68%), furosemide (2.63%), and clarithromycin (2.49%).

**Table 7:** The most reported drugs.

Drugs	Number	Percentage
Warfarin sodium	7649	4.32%
Aspirin	7434	4.20%
Sertraline hydrochloride	5744	3.24%
Tacrolimus	5476	3.09%
Simvastatin	5153	2.91%
Fluoxetine hydrochloride	5055	2.85%
Clopidogrel bisulfate	4744	2.68%
Furosemide	4654	2.63%
Clarithromycin	4419	2.49%
Venlafaxine hydrochloride	4257	2.40%
Amlodipine besylate	3958	2.23%
Omeprazole	3820	2.16%
Risperidone	3721	2.10%
Quetiapine	3663	2.07%
Warfarin	3546	2.00%
Methotrexate	3538	2.00%
Carbamazepine	3537	2.00%
Valproic acid	3408	1.92%
Lamotrigine	3389	1.91%
Metformin hydrochloride	3309	1.87%

The drugs that cause death commonly were diazepam (6.73%), aspirin (5.99%), acetaminophen (4.51%), sertraline (4.41%), methadone (3.99%), and alprazolam (3.83%). The drugs that cause disability commonly were metoclopramide (10.36%), simvastatin (7.75%), omeprazole (6.30%), clarithromycin (4.14%), sertraline (3.94%), and aspirin (3.79%). The drugs that caused hospitalization commonly were aspirin (5.45%), warfarin sodium (5.07%), furosemide (4.20%), simvastatin (3.97%), tacrolimus (3.72%), and clarithromycin (3.33%). The drugs that caused life-threatening consequences commonly were aspirin (5.54%), warfarin sodium (4.12%), furosemide (3.98%), simvastatin (3.81%), omeprazole (3.36%), and clopidogrel bisulfate (3.16%) (Table 8).

**Table 8:** The most reported drugs that cause serious.

Outcome	Drugs	Number and percentage
Died	Diazepam	998 (6.73%)
	Aspirin	888 (5.99%)
	Acetaminophen	669 (4.51%)
	Sertraline hydrochloride	653 (4.41%)
	Methadone hydrochloride	592 (3.99%)
	Alprazolam	567 (3.83%)
	Warfarin sodium	556 (3.75%)
	Clopidogrel bisulfate	530 (3.58%)
	Fluoxetine hydrochloride	523 (3.53%)
	Citalopram	503 (3.39%)
Disabled	Simvastatin	378 (7.75%)
	Omeprazole	307 (6.30%)
	Metoclopramide	274 (5.62%)
	Metoclopramide hydrochloride	231 (4.74%)
	Clarithromycin	202 (4.14%)
	Sertraline hydrochloride	192 (3.94%)
	Aspirin	185 (3.79%)
	Amlodipine besylate	153 (3.14%)
	Mirtazapine	143 (2.93%)
	Ciprofloxacin	143 (2.93%)
Hospitalized	Aspirin	4256 (5.45%)
	Warfarin sodium	3962 (5.07%)
	Furosemide	3279 (4.20%)
	Simvastatin	3100 (3.97%)
	Tacrolimus	2904 (3.72%)
	Clarithromycin	2604 (3.33%)
	Clopidogrel bisulfate	2578 (3.30%)
	Sertraline hydrochloride	2404 (3.08%)
	Fluoxetine hydrochloride	2323 (2.97%)
	Omeprazole	2083 (2.67%)
Life-threatening	Aspirin	909 (5.54%)
	Warfarin sodium	677 (4.12%)
	Furosemide	653 (3.98%)
	Simvastatin	625 (3.81%)
	Omeprazole	552 (3.36%)
	Clopidogrel bisulfate	519 (3.16%)
	Acetaminophen	517 (3.15%)
	Quetiapine	504 (3.07%)
	Metformin hydrochloride	490 (2.98%)
	Fluoxetine hydrochloride	483 (2.94%)

## 4. Discussion

The current study delves into the characterization of DDI reports lodged within the FAERS, illuminating the frequency and patterns of medications involved in these interactions. Through meticulous analysis, this research aims to unravel the complexities surrounding drug interactions, providing invaluable insights into their prevalence and potential implications for patient safety and pharmacovigilance endeavors. By examining the data within FAERS with a keen eye for detail, this study seeks to contribute to a deeper understanding of the landscape of drug interactions, ultimately facilitating more informed decision-making in clinical practice and drug regulatory processes.

The study findings revealed that individuals aged 18 to 64 comprised the majority, accounting for 52.61% of all DDIs, followed by those aged 65 to 85 years, who represented 36.66% of reports. As highlighted by Bories et al., older patients face an elevated risk of DDIs due to their multiple comorbidities, which often necessitate polypharmacy.^[18]^ Age-related changes in medication pharmacokinetics and pharmacodynamics further contribute to this heightened susceptibility to drug interactions. Additionally, research by Kim and Parish underscores that advanced age, along with the presence of multiple morbidities and polypharmacotherapy, are primary risk factors for adverse outcomes resulting from inappropriate medication combinations.^[19]^ Moreover, Hines and Murphy emphasize that elderly individuals are particularly vulnerable to drug interactions due to age-related physiological changes, increased susceptibility to age-related illnesses, and higher medication consumption rates.^[20]^ This distribution across age groups underscores the imperative to acknowledge and address medication interactions comprehensively, as they affect a significant proportion of the population. Recognizing and managing drug interactions across different age demographics is essential for optimizing patient safety and improving healthcare outcomes.

The fact that the majority of affected patients (52.81%) were females suggests potential gender-related variations in sensitivity to drug interactions or differences in prescription consumption habits. Research by Greaves et al has demonstrated that sex-related characteristics can influence pharmacokinetic/pharmacodynamic processes, as well as the occurrence and reporting of ADRs.^[21]^ Gender also plays a role in medication marketing and may influence demand, prescription practices, and the reporting of adverse events. Furthermore, according to Lapeyre-Mestre, being female is often associated with an increased likelihood of experiencing ADRs.^[22]^ Recent literature supports the notion that women may encounter different types of ADRs compared to men when treated with the same medications. Additionally, differences in ADR reporting between genders may be exacerbated by gender-related prescribing and management practices. This conclusion underscores the importance of incorporating gender-specific characteristics into pharmacovigilance and medication safety monitoring initiatives. By considering gender differences in drug response and ADR occurrence, healthcare professionals can better tailor treatment strategies to individual patient needs and improve overall medication safety and effectiveness.

Healthcare personnel played a pivotal role in identifying and reporting adverse medication interactions, contributing to 74.60% of all reports. The United States FDA actively encourages healthcare providers to report adverse events, medication errors, and concerns about product quality through the FAERS. This initiative aims to enhance post-market surveillance and bolster patient safety by uncovering previously unrecognized adverse events, medication interactions, and other safety issues associated with pharmaceutical products.^[23,24]^ The FDA underscores the crucial role of healthcare professionals in detecting and reporting these incidents, emphasizing their role as frontline guardians of patient safety. This underscores the importance of continuous education and training for healthcare personnel on medication safety and pharmacovigilance techniques. By equipping healthcare providers with the knowledge and skills needed to identify and report adverse events promptly and accurately, we can enhance early detection and intervention, thereby mitigating potential harm to patients and improving overall medication safety standards.

The study identified several frequently reported drugs involved in DDIs, including warfarin sodium, aspirin, sertraline hydrochloride, tacrolimus, and simvastatin. Warfarin, a medication with a narrow therapeutic index, is particularly prone to multiple interactions. Understanding the indications, mechanism of action, interactions, severe side effects, contraindications, toxicity, and monitoring protocols associated with warfarin is crucial for physicians leading patient management, especially when anticoagulation is recommended as part of an interprofessional team.^[25]^ Aspirin, another commonly implicated medication, is widely available and used for various therapeutic purposes. However, due to its potential side effects and interactions, all members of the interprofessional healthcare team need to be aware when patients are taking aspirin. Monitoring and counseling patients on aspirin use can help optimize therapeutic outcomes and prevent potential adverse effects of salicylic acid.^[26]^ By collaborating across disciplines and staying informed about medication profiles, healthcare professionals can ensure safe and effective patient care, particularly when managing medications prone to interactions and adverse effects like warfarin and aspirin.

Sertraline, an selective serotonin reuptake inhibitor, interacts with various medications. According to Low et al, the concurrent use of CYP2C9 inhibitors such as sertraline with sulfonylureas or nateglinide should be avoided due to the risk of hypoglycemia. Additionally, both NICE and the American Psychiatric Association recommend against using selective serotonin reuptake inhibitors in patients taking NSAIDs due to the increased risk of gastrointestinal bleeding.^[27]^ Tacrolimus, an immunosuppressive medication often used in transplantation, has its oral bioavailability affected by concurrent administration of CYP3A or P-glycoprotein inhibitors. Moreover, medications commonly used in transplantation, such as corticosteroids and antifungal agents, can influence tacrolimus concentrations by affecting liver CYP activity.^[28]^ Simvastatin, a cholesterol-lowering medication, is metabolized by the hepatic CYP450 enzyme system, particularly CYP3A4 and CYP2C6. This metabolism profile contributes significantly to some of its potential side effects and DDIs.^[29]^ Before initiating or discontinuing any medication, patients should consult a healthcare provider to be aware of potential drug interactions, particularly if taking multiple medications concurrently. Healthcare providers offer valuable information and advice on managing medication interactions to optimize treatment outcomes and prevent adverse effects. By promoting patient awareness and proactive communication with healthcare providers, potential drug interactions can be identified and managed effectively, ensuring patient safety and treatment efficacy.

The study findings highlight several medications, including diazepam, aspirin, acetaminophen, and sertraline, which were most frequently associated with fatalities. Metoclopramide, simvastatin, omeprazole, and clarithromycin were commonly linked to disabilities, while aspirin, warfarin sodium, furosemide, and simvastatin were prevalent causes of hospitalizations. Additionally, aspirin, warfarin sodium, furosemide, simvastatin, and omeprazole were identified as frequently causing life-threatening consequences. These findings underscore the critical importance of ongoing pharmacovigilance and proactive risk management strategies to minimize the potential harm associated with commonly prescribed medications. By fostering greater awareness among patients and healthcare professionals, we can work towards optimizing the safety and efficacy of pharmacotherapy and ultimately improving patient outcomes. Implementing robust monitoring systems and effective communication channels can help promptly identify and address adverse drug reactions and interactions, mitigating risks and enhancing patient safety throughout treatment.

The study acknowledged several limitations, including potential underreporting biases within the FAERS database, reliance on voluntary reporting, and the possibility of unaccounted confounding factors. Future research could thoroughly explore these limitations and employ additional methodologies to validate and complement the study’s findings. Moreover, variations in medication naming conventions, such as “aspirin” versus “acetylsalicylic acid” or “paracetamol” versus “acetaminophen,” could introduce inconsistencies in the reported data. Therefore, it is essential to carefully examine FAERS findings to identify the most frequently reported medications and standardize reporting practices to ensure accuracy and consistency in future analyses. By addressing these limitations and refining data collection and analysis methods, researchers can enhance the reliability and validity of pharmacovigilance studies, ultimately contributing to improved patient safety and healthcare outcomes.

## 5. Conclusions

The current study highlights the frequent reporting of drug interactions, with warfarin, aspirin, sertraline, tacrolimus, and simvastatin being among the most commonly reported medications. Healthcare practitioners play a crucial role in educating patients about potential adverse events, including drug interactions, and advising them on minimizing these risks. These findings carry significant implications for clinical practice, pharmacovigilance initiatives, and public health measures aimed at reducing the risks associated with drug interactions and enhancing patient safety. By increasing awareness among healthcare professionals and patients alike, proactive measures can be taken to prevent or mitigate the potential harms of drug interactions. This may include thorough medication reconciliation, regular monitoring for adverse effects, and clear communication between patients and healthcare providers regarding medication use and potential risks. Ultimately, prioritizing strategies to address drug interactions can improve patient outcomes and overall healthcare quality.

## Author contributions

**Conceptualization:** Nehad Ahmed.

**Data curation:** Nehad Ahmed.

**Formal analysis:** Nehad Ahmed.

**Funding acquisition:** Abdullah Alahmari.

**Investigation:** Nehad Ahmed.

**Methodology:** Nehad Ahmed.

**Project administration:** Abdullah Alahmari.

**Resources:** Sarah Fatani.

**Software:** Sarah Fatani.

**Supervision:** Abdullah Alahmari.

**Visualization:** Abdullah Alahmari.

**Validation:** Sarah Fatani.

**Writing – review & editing:** Sarah Fatani.

**Writing – original draft:** Nehad Ahmed.
